# Identification of quantitative trait loci associated with canopy temperature in soybean

**DOI:** 10.1038/s41598-020-74614-8

**Published:** 2020-10-19

**Authors:** Sumandeep K. Bazzer, Larry C. Purcell

**Affiliations:** Department of Crop, Soil, and Environmental Sciences, University of Arkansas, Fayetteville, AR 72704 USA

**Keywords:** Genetics, Plant sciences

## Abstract

A consistent risk for soybean (*Glycine max* L.) production is the impact of drought on growth and yield. Canopy temperature (CT) is an indirect measure of transpiration rate and stomatal conductance and may be valuable in distinguishing differences among genotypes in response to drought. The objective of this study was to map quantitative trait loci (QTLs) associated with CT using thermal infrared imaging in a population of recombinant inbred lines developed from a cross between KS4895 and Jackson. Heritability of CT was 35% when estimated across environments. QTL analysis identified 11 loci for CT distributed on eight chromosomes that individually explained between 4.6 and 12.3% of the phenotypic variation. The locus on Gm11 was identified in two individual environments and across environments and explained the highest proportion of phenotypic variation (9.3% to 11.5%) in CT. Several of these CT loci coincided with the genomic regions from previous studies associated with canopy wilting, canopy temperature, water use efficiency, and other morpho-physiological traits related with drought tolerance. Candidate genes with biological function related to transpiration, root development, and signal transduction underlie these putative CT loci. These genomic regions may be important resources in soybean breeding programs to improve tolerance to drought.

## Introduction

Soybean (*Glycine max* (L.) Merr.) is one of the most important crops grown in the US on an area of 30.8 million hectares with a production of 96.8 million metric tons and that contributes around 28% to global production (www.soystats.com). The sustainability of soybean production is threatened by climate changes such as increased temperature, erratic precipitation, and variable weather patterns. Among these factors, drought is one of the major constraints limiting yield potential in legumes and other cereal crops^1–5^. Various field studies had reported that drought stress leads to the reduction of 5 to 50% of soybean yield^6,7^. Thus, there is need for development of cultivars with drought tolerance to cope with adverse climatic conditions and to improve crop performance. Selection for high yield is difficult under drought stress conditions due to its quantitative nature and because of a high interaction of genotype with the environment^8^. Therefore, it is important to identify specific physiological traits that may improve the crop performance and yield under water deficit conditions.

Canopy temperature can be used as surrogate measurement of plant water balance/relations and is an important physiological trait associated with drought tolerance^9–15^. Canopy temperature is closely associated with transpiration rate and stomatal conductance in many crops^13,16^. Due to evaporative cooling, transpiration is negatively correlated with canopy temperature^17^. Under optimum moisture conditions, increased vapor pressure deficit increases evaporative demand resulting in higher transpiration rate and a decrease in canopy temperature provided that stomatal conductance does not change. However, decreased transpiration rate and stomatal conductance under water deficit conditions limits evaporative cooling and leads to increased canopy temperature^18–20^. The genotypes with a cooler canopy under water deficit condition continue transpiration at a relatively high rate, presumably due to a larger store of soil available water compared to genotypes with higher canopy temperature^21–23^.

In bread wheat (*Triticum aestivum*) and durum wheat (*Triticum durum*), there is a significant correlation between cooler canopy temperature high yield^15,24,25^. Likewise, there were significant genetic gains in wheat yield when direct selection was made based on a cooler canopy^23,26,27^. A significant correlation between canopy temperature and transpiration was found in sugar beet (*Beta vulgaris*), rice (*Oryza sativa*), and potatoes (*Solanum tuberosum*)^28^. Cooler canopy (or canopy temperature depression) was positively correlated with grain yield in rice^29^, sugarcane (*Saccharum officinarum*)^10,30^, and chickpea (*Cicer arietinum*)^31,32^, pearl millet (*Penniserum americanum*)^33^, and soybean^34^ under water deficit conditions. Canopy temperature depression (CTD) is defined as deviation of plant canopy temperature from the air temperature. Bai and Purcell^9^ found that slow wilting soybean genotypes under drought had a cooler canopy than fast wilting genotypes and that a cooler canopy was positively associated with grain yield.

Manual phenotyping/measurement of transpiration and stomatal conductance to detect canopy temperatures differences is difficult and tedious. Therefore, selection criteria for genotypes with cooler canopy must be rapid, relatively simple, and allow the screening of large number of field plots in a short period of time^35,36^. The advent of high throughput phenotyping platforms has led to rapid, accurate, and non-destructive monitoring of whole-plant responses and differences in stomatal behavior to water stress^37–40^. Unmanned aerial systems (UAS) provide an efficient phenotyping platform to evaluate a large number of experimental fields for precise, quantitative assessment of CT in segregating mapping populations, and allowing a comparison among genotypes for CT differences^13^. Thermal infrared imaging from a UAS has become an important tool in early growth stages to detect drought stress, improve water use efficiency (WUE), and precisely manage irrigation^12,41–44^.

Combining thermal infrared imaging with genetic mapping may help in understanding the genetic architecture of drought tolerance^45–49^. Mapping studies for CT have been reported in wheat^16^, rice^50^, and maize (*Zea mays*)^51^. In soybean, genome wide association studies (GWAS) and linkage mapping studies have dissected the genetic basic of several morpho-physiological traits such as canopy wilting^52–56^, carbon isotope ratio (δ^13^C)^57–60^, oxygen isotope ratio (δ^15^O)^60^, and canopy coverage^61^. The first GWAS analysis for CT in soybean was conducted using a diverse panel of 345 maturity group IV accessions^14^. Association analysis identified 34 loci associated with CT. However, to date, there has been no report of linkage mapping using thermal infrared imaging to study the genetic basis of CT in soybean.

Thus, the present study aimed to identify the genomic regions associated with CT using a mapping population of 168 recombinant inbred lines (RILs) developed from a cross between KS4895 and Jackson. The objectives of this study were to (1) identify QTLs associated with CT (2) confirm identified CT QTLs with previously mapped QTLs associated with drought tolerance; and (3) search for putative candidate genes underlying these QTLs.

## Materials and methods

One hundred and sixty-eight F_5_-derived recombinant inbred lines (RILs) generated from a cross between KS4895 (PI 595081)^62^ and Jackson (PI 548657)^63^ were used to identify genomic regions associated with CT. KS4895 is a maturity group (MG) IV cultivar developed by the Kansas Agricultural Experiment Station, and Jackson is a MG VII cultivar developed by the North Carolina Agricultural Experiment Station and the USDA-ARS. These two genotypes were selected as parents because previous research reported that Jackson was more tolerant than KS4895 with regards to N_2_ fixation response to drought^64,65^. Populations developed from a cross of KS4895 and Jackson were used to study genetic control of traits associated with N_2_ fixation^66^, canopy wilting^54,55^ and δ^13^C^58^.

The RIL population along with parents were evaluated for CT at the Pine Tree Research Station, AR (35.11° N, 90.91° W) on a Calloway silt loam soil (fine, montmorillonitic, thermic Typic Albaqualfs) and Rohwer Research Station, AR (33.80° N, 91.28° W) on a Sharkey silty clay soil (very-fine, smectitic, thermic Chromic Epiaquerts) in three consecutive years (2017–2019). Each location by year combination was treated as an individual environment and designated as PT17 (Pine Tree 2017), RH17 (Rohwer 2017), PT18 (Pine Tree 2018), RH18 (Rohwer 2018), PT19 (Pine Tree 2019), and RH19 (Rohwer 2019). A randomized complete block experimental design with two replications was employed at each environment. The details of planting date, CT measurement date, and weather data on the CT measurement day are presented in Table 1. The RILs were planted in nine-row plots that were 4.0–4.5 m long with 0.15–0.18 m spacing between rows. The average minimum temperature, maximum temperature, and rainfall for Pine Tree and Rohwer in 2017, 2018, and 2019 were collected from the Southern Regional Climate Center (www.srcc.lsu.edu/station_search) using the Climate Data Portal. An irrigation scheduling program^67^ was used to estimate soil moisture deficit. Vapor pressure deficit (VPD) for each environment was estimated from daily maximum and minimum temperatures, assuming that water vapor pressure was saturated at the minimum temperature^68,69^. The experiments were rainfed for all environments, and experimental fields were managed using recommended practices.

**Table 1:** Planting date and weather data including maximum temperature (MaxT), minimum temperature (MinT), No. of days without rainfall, estimated vapor pressure deficit (VPD), and estimated soil moisture deficit at the time of canopy temperature measurements for the field experiments conducted at Pine Tree (PT) and Rohwer (RH) in 2017, 2018, and 2019.

Env.	Planting date	CT recording date	MaxT (°C)	MinT (°C)	No. of days	VPD (kPa)	Soil moist. deficit (mm)	h^2b^ (%)
PT17	9 June 2017	25 Aug 2017	29	15	8	2.3	49	9
RH17	8 June 2017	21 July 2017	34	25	4	2.3	50	22
PT18	7 June 2018	25 July 2018	33	19	8	2.9	> 75	11
RH18	31 May 2018	19 July 2018	31	24	11	1.6	71	7
PT19	31 May 2019	10 Sept 2019	37	22	13	3.5	> 75	51
RH19	12 June 2019	9 Sept 2019	36	21	13	3.3	> 75	62
AE^a^	–	–	33	21	57	2.6	66	35

^a^AE denote averaged across environments.

^b^h^2^ narrow sense heritability.

### Phenotypic evaluation

CT measurements were made multiple times (two to five times) in all environments between 1200 and 1400 h when the sun was unobstructed by clouds. All measurement dates occurred once the canopy was completely closed to avoid the CT measurements being overwhelmed by the heat signature from the soil that occurred prior to canopy closure. Dates for CT measurement were targeted when the estimated soil moisture deficit was > 45 mm^67^ and soybean development was between full bloom and beginning seedfill^9,14^. Data ultimately used for QTL analysis for each environment were chosen for those dates in which the estimated soil moisture deficit was the greatest as previous research had determined that genotypic differences in CT were not apparent under water replete conditions^9^.

Canopy temperature was determined by using aerial infrared image analysis. A drone (DJI Phantom 4 Pro, www.dji.com/phantom-4-pro) equipped with an infrared (IR) camera (Model: FLIR Tau 2, www.flir.com) with a sensor size of 640 × 512 pixels, 25 mm focal length, sensitivity of 0.05 °C, and an accuracy of ± 5%, was flown at a height of 120 m above the ground at full canopy coverage. A digital video recorder recorded the video stream from the camera. The IR camera is not calibrated. That is, values of CT from the IR range from 0 (cool) to 255 (hot) and cover a range of 12.5 °C, but the specific temperature of the canopy is not determined. Herein, we report values directly from the IR camera as a measure of CT.

The IR images were extracted from the video file using VLC video player (www.videolan.org), and 6 to 17 images representing each plot multiple times were selected manually. Selected IR images were processed using FieldAnalyzer software (www.turfanalyzer.com/field-analyzer) to extract CT values from the average values of 400 to 2000 pixels from the center portion of the IR image of each plot and was used as a measure of CT. There were multiple CT values of each plot extracted from multiple selected images. The final CT values used for analysis was the average CT values determined from analyzing multiple images and after removing values that were more than ± 2 standard errors from the mean.

### Statistical analysis

The phenotypic analysis of CT was performed using SAS v.9.2 software (SAS Institute, 2013). The normality of CT in an individual environment was checked using a Q–Q plot of residuals and the Shapiro–Wilk test^70^. The presence of statistical differences between parents for CT was estimated using a *t* test. Pearson’s correlation analysis between environments and analysis of variance (ANOVA) averaged over environments were performed using PROC CORR (*α* = 0.05) and PROC MIXED procedures, respectively. Heritability was estimated from the variance components calculated with restricted maximum likelihood (REML) method of VARCOMP procedure. Narrow sense heritability (h^2^) was calculated using the following formula^71^:

$${\text{Across}}\;{\text{environments:}}\;{\text{h}}^{{2}} = \frac{{\sigma_{G}^{2} }}{{\sigma_{G}^{2} + \left( {\frac{{\sigma_{GE}^{2} }}{E}} \right) + \left( {\frac{{\sigma_{e}^{2} }}{RE}} \right)}}$$

$${\text{Within}}\;{\text{environments:}}\;{\text{h}}^{{2}} = \frac{{\sigma_{G}^{2} }}{{\sigma_{G}^{2} + \left( {\frac{{\sigma_{e}^{2} }}{R}} \right)}}$$

where $\sigma_{G}^{2} ,$ $\sigma_{GE}^{2} ,$ $\sigma_{e}^{2}$ are genotypic variance, genotype-by-environment variance, and error variance, respectively, and *E* and *R* are the number of environments and replications, respectively. Because F_5_-derived RILs were used in this research, $\sigma_{G}^{2}$ was composed entirely of additive variance and additive × additive epistasis variance, with negligible variance associated with other components of dominance variance. As the result, this heritability should be considered as narrow sense heritability. BLUPs (best linear unbiased predictions) were calculated using PROC MIXED procedure for individual environments and averaged across environments, considering all factors in the model as random. Environment was considered a fixed effect in the combined data analysis. QTL analysis of CT was performed using BLUP values to reduce the environmental variations.

### QTL analysis

The genetic map for the KS4895 × Jackson mapping population was previously described by Hwang et al.^54^ and used for QTL analysis in the present study. Briefly, the linkage map consists of 38 simple-sequence repeat (SSR) and 510 single-nucleotide polymorphism (SNP) markers with a map size of 2089 cM. The list of markers and their physical position on specific chromosomes is provided in Supplementary file 1. The BLUP estimates calculated for individual environments and averaged across environments were used for QTL analysis. The QTL analysis was performed with WinQTL Cartographer v.2.5^72^ using composite interval mapping (CIM) and multiple interval mapping (MIM) methods. CIM was performed using Model 6 (standard model) of the *Zmapqtl* program module^73^. Markers as co-factors to control background noise were selected with forward and backward stepwise regression methods with a walk speed and window size of 1 cM. A significant LOD (log of odds) threshold score was determined by a permutation test with 1000 runs and with a genome wide type I error of 5%^74^. The most likely position of QTLs and an estimate of the magnitude of their additive effects were determined using the CIM method^75^. The confidence interval for putative QTL positions was determined by one-LOD drop on either side of the LOD peak.

Multiple interval mapping (MIM) is a stepwise model procedure^76^ in which presence of significant QTLs and QTL × QTL interactions are detected using QTLs identified in the CIM method as an initial MIM selection model. This pre-selected MIM model was optimized for identified QTLs, search for new QTLs, and QTL × QTL interactions by using the ‘optimize QTLs positions’, ‘search for new QTLs’, and ‘QTL interactions’ options, respectively. The MIM model was determined with the minimum Bayesian information criterion (BIC), *c*(*n*) = *In*(*n*), and with genome walk speed and window size of 1 cM. The criterion used to declare coincident QTLs between environments was based on at least a 10 cM overlap in QTL intervals on the linkage map. In this study, a QTL that explained more than 10% of phenotypic variation was considered a major QTL.

### Candidate genes identification

Candidate genes for genomic regions underlying putative QTLs for CT identified in each environment and across environments were searched using the genome browser of Soybase (www.soybase.com). The candidate genes falling within ± 1 MB from the nearest marker of putative QTLs were selected according to the *Glyma2.0* assembly in Soybase (www.soybase.org) with consideration for those genes having biological function related to transpiration, canopy temperature, rooting, and plant water relations.

## Results

### Phenotypic evaluation

On the dates for CT measurement, the maximum temperature ranged from 29 (PT17) to 37 °C (PT19) and minimum temperature ranged from 15 (PT17) to 25 °C (RH17) (Table 1). The estimated VPD was ≥ 2.3 kPa on day of CT measurements in all environments except for RH18 in which VPD was 1.6 kPa. There had been no rainfall from 4 to 13 days prior to CT measurements. The estimated soil moisture deficit ranged from 49 mm to more than 75 mm on the day of CT measurements. Irrigation is recommended if estimated soil moisture deficit exceeds 37 mm for silt loam soils present at Pine Tree and 50 mm for silty clay soils present at Rohwer^67^, indicating drought–stress conditions on the day of CT measurements.

**Figure 1:**
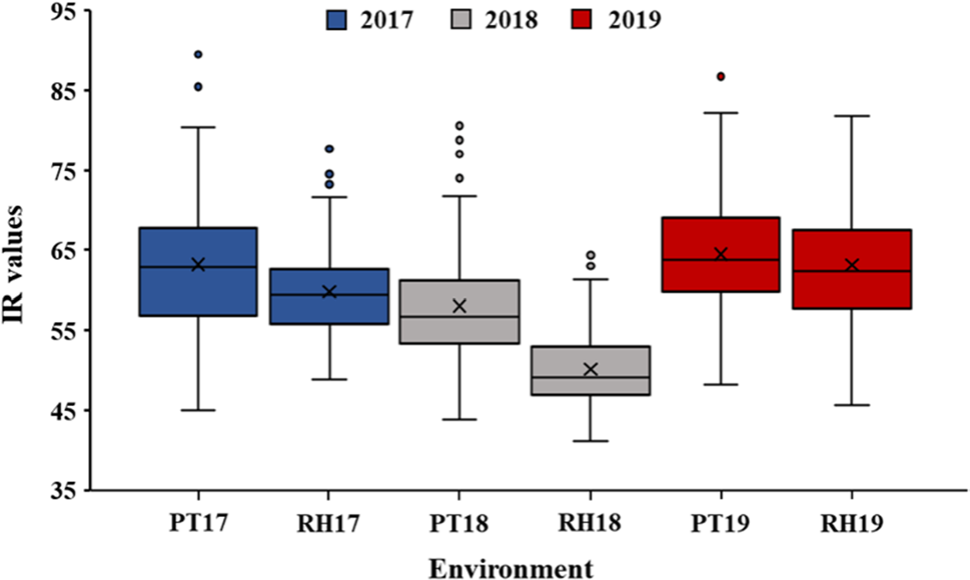
The box plots showed a broad range of canopy temperature in KS4895 × Jackson RIL population within each environment. Environment prefixes PT and RH denotes Pine Tree and Rohwer, respectively followed by 17 (2017), 18 (2018), and 19 (2019) for years. Box edges represent the upper and lower quartile with a median (bold line in the middle of box) and mean value (cross in the middle of box).

The CT values had a wide range in all environments with RIL means that ranged from 50 to 64 (Fig. 1, Table 2). Jackson had lower CT values than KS4895 in all environments except RH17 (Table 2), and parents were significantly (*P* < 0.05) different in PT17, RH19, and across environments (AE) (Table 2). Averaged over environments, IR values were 12 units less for Jackson than KS4895, which indicate a CT that was approximately 0.59 °C cooler for Jackson. The distributions of CT values were approximately normal (*P* < 0.001) except for PT18 and RH18, which were slightly skewed left as indicated by the Shapiro and Wilk test (data not shown, Fig. 1). There were weakly significant correlations for CT between environments for PT17 and PT18 (*r* = 0.18*), PT17 and RH19 (*r* = 0.26**), RH17 and RH19 (*r* = 0.15*), PT19 and RH18 (*r* =  − 0.16*), and PT19 and RH19 (*r* = 0.49***). Across environments, ANOVA indicated that there was a significant effect (*P* < 0.05) of RILs, environment, and RILs × environment interaction on CT, indicating that CT among RILs was affected differently by environmental conditions (data not shown). The narrow sense heritability of CT for individual environments was 9% (PT17), 11% (PT18), 51% (PT19), 22% (RH17), 7% (RH18), and 62% (RH19) and was 35% when estimated across environments (Table 1).

**Table 2:** A summary statistic of canopy temperature (CT) in the parents (KS4895 and Jackson) and RILs (n = 168) population of KS4895 and Jackson evaluated at Pine Tree and Rohwer in 2017, 2018 and 2019.

Trait	Env.^a^	Parental means		RILs population	
		KS4895	Jackson	Mean	Range^c^
CT	PT17	70*	59	63	45
	RH17	57	67	60	29
	PT18	71	54	58	37
	RH18	59	47	50	24
	PT19	71	63	64	39
	RH19	86*	53	63	36
	AE^b^	69*	57	60	35

*Indicated significant difference (*P* ≤ 0.05) between parents.

^a^Environments: Prefixes PT and RH denotes Pine Tree and Rohwer, respectively followed by 17 (2017), 18 (2018), and 19 (2019) for years.

^b^AE denotes averaged across environments.

^c^Range of RIL population is the difference between maximum and minimum IR values.

### QTL analysis

Analysis of QTLs associated with CT in individual environments identified seven QTLs present on five chromosomes using the CIM method. Of these seven QTLs, one QTL was identified in RH17, PT18, and RH18 and two QTLs were identified in PT19 and RH19. No QTLs were detected in PT17. These QTLs had additive effects that ranged from − 0.08 to − 1.17 and explained 5.7% to 12.3% of the phenotypic variation (Table 3). The QTLs identified in PT18 (1), PT19 (2), RH18 (1), and RH19 (1) were also identified by the MIM method. Two additional QTLs present on Gm13 (at 31355907 bp) in RH18 and on Gm16 (at 26745906 bp) in PT19 were identified by the MIM method but were not identified by the CIM method (Table 3).

**Table 3:** The QTLs associated with canopy temperature identified by composite interval mapping (CIM) and multiple interval mapping (MIM) in a RIL population of KS4895 and Jackson which were evaluated at Pine Tree and Rohwer in 2017, 2018, and 2019.

Locus	Chrom.^a^	Env.^b^	Position (bp)^c^	Nearest marker^d^	Additive effect^e^	R^2f.^	Favorable allele^g^	FM at 95% CI^h^	Method
1	Gm02	PT18	2175799	ss107919808	− 0.27	12.3	KS4895	ss107919971-ss107912545	CIM, MIM
2		AE	3162143	ss107912545	− 0.42	6.3	KS4895	ss107919808-ss107913715	CIM, MIM
3	Gm03	RH18	3847841	ss107929820	− 0.08	6.5	KS4895	ss107913533-ss107912527	CIM, MIM
4	Gm10	PT19	2453116	ss107921662	1.19	8.5	Jackson	ss107921662-ss107930841	CIM, MIM
5	Gm11	PT19	10350509	ss107919087	− 1.28	11.5	KS4895	ss107919087-ss107913812	CIM, MIM
		RH19	10350509	ss107919087	− 1.47	10.8	KS4895	Satt197-ss107927406	CIM, MIM
		AE	10350509	ss107919087	− 0.51	9.3	KS4895	Satt197-ss107927406	CIM, MIM
6	Gm13	RH18	31355907	ss107912665	− 0.07	4.6	KS4895	ss107915606-ss107912922	MIM
7	Gm15	AE	51379618	ss107925861	− 0.42	5.4	KS4895	ss107914616-Sat_376	MIM
8	Gm16	PT19	26745906	ss107927055	0.65	4.9	Jackson	ss107927440-ss107913908	MIM
9	Gm18	AE	17323638	ss107921048	− 0.39	5.3	KS4895	ss107920369-ss107914987	CIM
10	Gm18	RH17	50727159	ss107913405	− 0.27	5.7	KS4895	ss107919708-ss107921608	CIM
		AE	50727159	ss107913405	− 0.40	5.4	KS4895	ss107919708-ss107913107	CIM
11	Gm18	RH19	56161047	ss107929216	− 1.29	8.8	KS4895	ss107929175-ss107919550	CIM
		AE	56161047	ss107929216	− 0.41	6.2	KS4895	ss107929175-ss107919550	CIM, MIM

^a^*Glycine max* chromosome on which QTL was present.

^b^Environment in which specific QTL was identified. Prefixes PT and RH denotes Pine Tree and Rohwer, respectively followed by 17 (2017), 18 (2018), and 19 (2019) for years. AE denotes averaged across environments.

^c^QTL position in base pairs on respective chromosomes according to *Glycine max* genome assembly 2.0 (Glyma.Wm82.a2; www.soybase.com).

^d^Nearest marker to the QTL.

^e^Additive effect of the QTL.

^f^Proportion (%) of phenotypic variation explained by specific QTL.

^g^Allele that decreases CT values was considered as the favorable allele; Positive sign indicates that favorable alleles (decreasing CT) were from Jackson and negative sign indicates the KS4895 allele.

^h^Flanking markers (FM) present near or at 95% confidence interval (CI) of the maximum likely QTL positions. The LOD values with ± 1 declination was used to estimate the 95% confidence interval.

Across environment (AE) QTL analysis of CT identified five QTLs present on Gm02 (1), Gm11 (1), and Gm18 (3) with additive effects ranging from − 0.39 to − 0.51 and explaining phenotypic variation from 5.3 to 9.3%. Three out of five AE QTLs were common between CIM and MIM methods, and one new QTL on Gm15 (at 51379618 bp) was identified only by the MIM method (Table 3). The QTL present on Gm11 (at 10350509 bp) was common among PT19, RH19, and AE. The QTL on Gm18 (at 50727159 bp) was identified in RH17 and AE, and the QTL on Gm18 (at 56161047 bp) was common for RH19 and AE. All other QTLs were environmentally specific (Table 3). The favorable alleles for deceasing CT for all the QTLs were from KS4895 except for the QTLs on Gm10 and Gm16. QTL × QTL interactions were identified between the QTLs present on Gm11 and Gm16 in PT19 by the MIM method. This interaction explained 5.7% of the phenotypic variation with the favorable allele contributed by KS4895.

### Candidate gene identification

There were more than 1092 candidate genes present within ± 1 MB of the nearest markers for putative QTLs with a range from 53 to 206 genes for individual QTLs. A list of these genes (*Glyma2.0 ID*) with their biological annotation is provided in Supplementary file 2. Out of these 1092 genes, those having biological function related to stomatal complex morphogenesis, regulation of stomatal movement, response to water deprivation, response to abscisic acid (ABA), ABA mediated signaling pathway, ABA transport, root hair elongation, root hair cell differentiation, primary and adventitious root development, root morphogenesis, water transport, response to osmotic and oxidative stress, signal transduction, and response to different hormones stimulus were considered to play a potential role in controlling CT in response to different soil moisture conditions (bold text in Supplementary file 2).

## Discussion

Remote sensing with thermal infrared imagining provides a powerful approach to measure and compare temperature differences of plant canopies of a large number of genotypes for field scale experiments and has been widely used in various crops^3,13–15,43,77^. The present study investigated the genetic control of CT in a population derived from a cross between KS4895 and Jackson, which was evaluated across six environments. Under replete soil moisture and aerial environmental conditions, plants continue to transpire through open stomata. In contrast, as soil moisture becomes limiting, plants close stomata as a preventive mechanism, transpiration decreases, and there is an increase in CT^31^. Those genotypes that have access to soil moisture continue transpiration during drought stress, resulting in a cooler canopy. High soil moisture deficit and VPD at the time of CT measurements resulted in drought stress in all environments (Table 1). The PT19 and RH19 environments had greater soil moisture deficit and VPD, resulting in greater differences among RILs and higher heritability of CT as compared to other environments.

For all environments there was transgressive segregation among RILs with lower and greater CT than the parents, indicating the distribution of favorable alleles for cooler canopy temperature in both parents. CT is highly influenced by environmental conditions (soil moisture availability, vapor pressure deficit, air temperature) and plant morphology (canopy and root architecture conditions)^34,78^, resulting in significant genotype × environment interactions. While the h^2^ of CT was 35% when estimated across six environments, and the range of h^2^ among environments was from 7 to 62% (Table 1). The two environments in which h^2^ was > 50% were noted for a severe soil moisture deficit (> 75 mm), maximum temperatures ≥ 36 °C, and VPD ≥ 3.3 (Table 1). From this it appears that high h^2^ did not appear to be solely due to soil moisture deficit as PT18 and RH18 had severe soil moisture deficit but had h^2^ ≤ 11%. Environments with high h^2^ (PT19 and RH19) both had severe soil moisture deficit (≥ 75 mm) combined with high temperature (≥ 36 °C) and high VPD (≥ 3.3 kPa). Along with making measurements when the canopy is completely closed and the sun is completely unobstructed, other conditions for increasing h^2^ for CT include making measurements when there is a high soil moisture deficit, VPD exceeds 3.3 kPa, and when maximum temperatures are high. Additional research is needed in order to define better the conditions that would optimize CT measurements and increase h^2^. Kaler et al.^14^ reported a broad sense heritability (H) of 19% for CT in a diverse panel of soybean accessions evaluated in different environments using GWAS. Likewise, low to moderate broad sense heritability of CT/CTD was reported in wheat (H = 16% to 38%)^79–81^, in sugarcane (H = 9% to 44%)^82^, and in rice (H = 21% to 30%)^83^. The low heritability of CT/CTD in soybean and other crops indicate that this trait is highly influenced by environmental conditions^84^.

The complexity of CT in soybean is further indicated by detection of multiple QTLs by CIM and MIM methods. The QTL analysis for CT using CIM and MIM methods identified a total of nine QTLs in individual environments and six QTLs when data were averaged across environments (Table 3). Most of these QTLs were environmentally specific except the QTLs present on Gm11 (at 10350509 bp) and Gm18 (at 50727159 bp and 56161047 bp), which were common in at least one individual environment and across environments (Table 3). The QTLs present at Locus 5 on Gm11 explained phenotypic variation that ranged from 9.3% to 11.5% and is considered a major QTL (Table 3). The markers linked with these QTLs have potential utility using marker assisted selection or genomic selection in a breeding program. The inconsistency of QTLs among environments might be due to different environmental factors such as field moisture status, soil temperature and depth, solar radiation, and VPD. Although QTLs were generally environmentally specific, most of these QTLs were detected by both CIM and MIM methods, which increases the confidence of these results. Of particular interest are environmental conditions that could improve consistency and increase heritability of CT. More research is needed to increase the utility of markers linked with QTLs identified in specific environments in selecting genotypes with a cooler canopy and to determine environmental conditions that optimize heritability.

**Figure 2:**
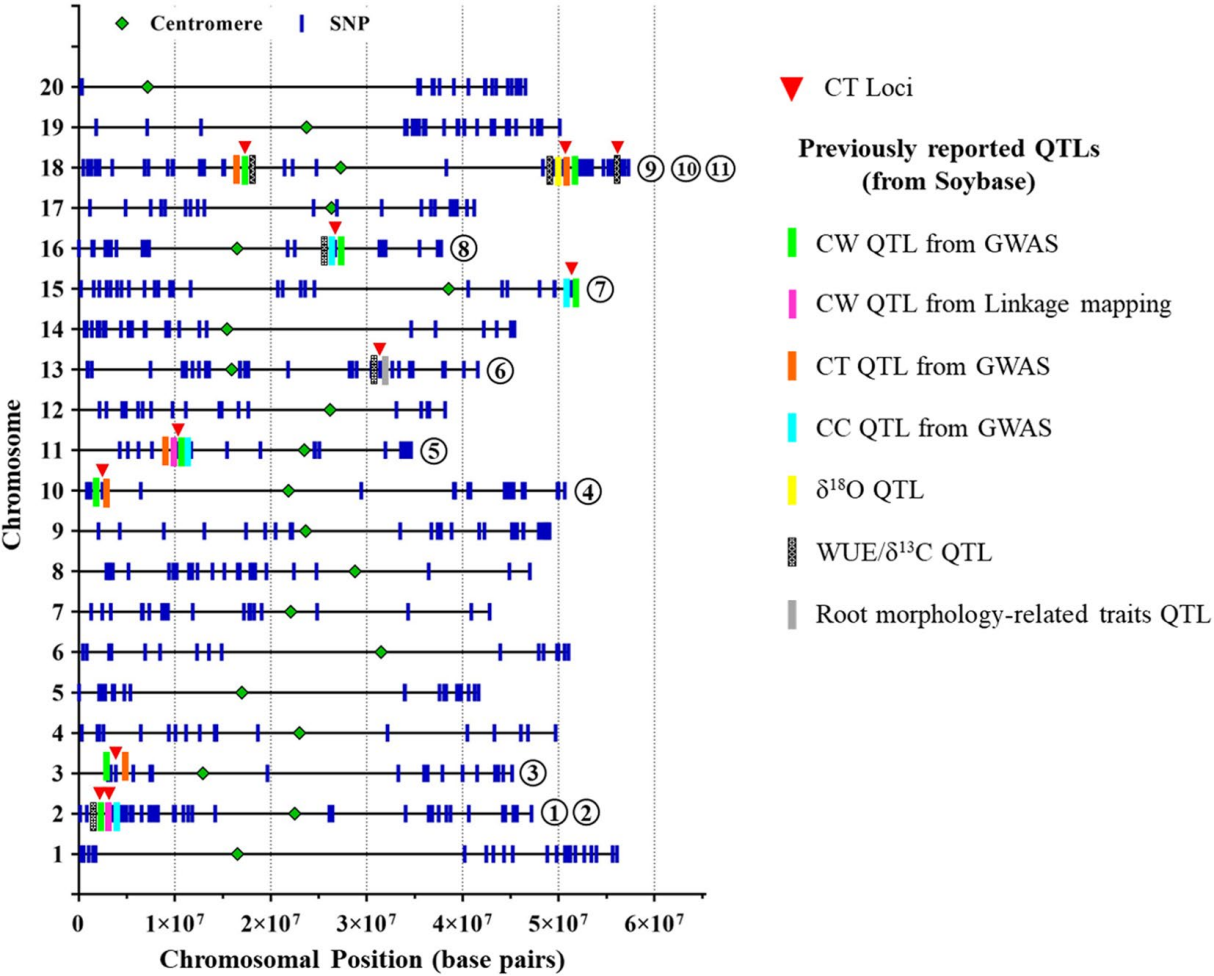
Physical position of SNPs on soybean chromosomes and position of loci (red downward triangle) associated with CT. The numbers in the black circles represent the loci number on a specific chromosome. Vertical colored bars (except blue) indicate the other QTLs found at the same positions in previous studies. CW and CT denote canopy wilting and canopy temperature, respectively.

Considering the overlapping confidence interval of QTLs identified in individual environments and across environments, we found 11 loci on eight chromosomes (Table 3, Fig. 2). The flanking markers position of each loci is provided in the Supplementary file 3. Even though Jackson tended to have lower CT among environments (Table 2), there were nine favorable loci from KS4895 and two favorable loci from Jackson. Hwang et al.^54^ found that KS4895 contributed many of the favorable alleles for slow canopy wilting that were identified in multiple biparental populations. Bai and Purcell^9^ found a positive correlation (0.37 < *r* < 0.62) between cooler CT and slow canopy wilting. There is a possibility that KS4895 has both slow canopy wilting and cooler canopy temperature alleles in the KS4895 × Jackson population.

For comparative analysis of CT loci with QTLs associated with plant water relations and drought tolerance related traits, the putative CT loci were aligned on the soybean linkage map in Soybase (www.soybase.com). The QTLs previously mapped within the 95% confidence interval of putative QTLs in this study were considered to be present in the same genomic regions. The CT Loci 1 and 2 (on Gm02) coincide with a canopy wilting QTL found in a population derived from KS4895 and Jackson (09705KJ population) that is different from the population used in the present research and in a population derived from Benning and PI 416937^54^. The favorable alleles for slow canopy wilting at this locus are from KS4895 and PI 416937, consistent with the present research that the KS4895 allele is associated with cool CT. These loci localized in a genomic cluster found in meta-analysis of canopy wilting QTLs using multiple biparental populations^55^. The loci were also mapped in an association panel in the genomic regions associated with canopy wilting^56^, canopy coverage^61^, and δ^13^C^60^. The co-localization of these drought-related QTLs with CT indicates the strong relationship among transpiration, WUE, canopy wilting, and CT.

Locus 3 (on Gm03) and Locus 4 (on Gm10) coincide with QTLs for CT^14^ and canopy wilting^56^, respectively, that were identified in GWAS. Locus 5 (on Gm11) maps to the same genomic region associated with CT^14^, canopy wilting^56^, and canopy coverage^61^ identified in GWAS analysis conducted using a diverse panel of soybean accessions. Locus 5 also coincides with a QTL for canopy wilting^54^ and for yield (unpublished results, Bazzer and Purcell) identified in population with KS4895 as a parent; the favorable alleles for slow canopy wilting in both these populations were from KS4895 as they were for CT in the present study.

Locus 6 (on Gm13) overlaps with a QTL for δ^13^C found by GWAS^60^. Nguyen et al.^85^ mapped a QTL for root area and root length at the same genomic location in a population derived from Tachinagaha × Iyodaizu. Rooting depth and area are drought avoidance mechanisms that may increase water availability^86^. The coincidence of CT and root morphology QTLs may point to a root system that extracts water from deeper soil horizons and results in cooler canopy during drought. In wheat, CT QTLs have been linked with rooting traits that allow extraction of more water from soil under drought^87^.

Locus 7 (on Gm15) and Locus 8 (on Gm16) coincide with canopy wilting^56^ and canopy coverage^61^ that were identified by GWAS. In addition, Locus 8 also overlaps with δ^13^C^60^. Earlier canopy coverage helps to decrease the water loss by soil evaporation relative to transpiration and improve WUE^88^. Locus 9 (on Gm18 at 17323638 bp) coincides with a WUE QTL mapped in a Young × PI416937 population with the ‘Young’ allele increasing WUE^89^. Locus 9 and Locus 10 (on Gm18 at 50727159 bp) also fall in the genomic region harboring QTLs for CT^14^, canopy wilting^56^, and δ^13^C^59,60^ identified by GWAS. In addition, Locus 10 overlaps with the oxygen isotope ratio^60^, which is a proxy for measurement of transpiration and is associated with stomatal conductance^90^.

Locus 11 (on Gm18 at 56161047 bp) coincides with δ^13^C identified in the same population as the present research^58^. The favorable allele for δ^13^C at this locus was from Jackson, while KS4895 provided the favorable allele for CT at this locus. The coincidence of δ^13^C and CT QTLs illustrates a shared genetic relationship between these two physiological traits. The co-localization of putative CT loci with QTLs associated with other morpho-physiological traits such as WUE, canopy wilting, canopy coverage, and transpiration may be a pleiotropic effect of the same genes controlling these traits or that the genes are spaced closely together on specific chromosomes.

The candidate gene search underlying putative CT loci identified genes with functions related to plant water relations, root morphology, and transpiration. These include genes involved in stomatal complex morphogenesis, regulation of stomatal movement, response to water deprivation, response to abscisic acid (ABA), ABA mediated signaling pathway, ABA transport, root hair elongation, root hair cell differentiation, response to oxidative stress, and signal transduction.

The upregulation of root morphology (lateral root formation, root hair elongation, root development response to ABA) related genes during drought may result in extracting residual soil moisture that maintains primary growth and developmental processes. In wheat, deeper root development in response to drought stress resulted in a cooler canopy and an increase in yield^25,91^. Aquaporin-related genes were also found underlying CT loci and these membrane proteins allow movement of water throughout plant in response to stress^92^. The co-localization of CT loci with QTLs associated with drought tolerance related traits and with underlying candidate genes with biological function related to transpiration, stomatal conductance, and plant water relations increases the probability that putative CT loci are associated with variation in CT in the present research. Additional research is needed to confirm the canopy temperature QTLs in different populations and in different environments to increase the efficiency of these genomic regions in selecting genotypes with a cooler canopy and with drought tolerance.

## Conclusions

In the present study, we identified genomic regions associated with CT in a recombinant inbred population derived from KS4895 and Jackson that was phenotyped in six different environments. These results represent the first QTLs for CT identified in soybean using a biparental population. The population segregated for CT in all environments and was used for QTL analysis. The heritability of CT was relatively low as compared to other morpho-physiological traits due to greater influence of different environmental factors. Eleven genomic loci present on eight chromosomes for CT were identified across several environments. The CT locus present on Gm11 explained phenotypic variation more than 10% and was considered as a major QTL. The favorable allele for cooler canopy for most of the loci were from KS4895, which were also coincident with canopy wilting QTLs identified in multiple biparental populations by Hwang et al.^54^. The identified CT QTLs coincided with QTLs associated with drought tolerance related traits mapped in previous studies, and genomic regions underlying these putative CT have the genes with biological function related to transpiration, stomatal conductance, and plant water relations. More research is needed to confirm these QTLs in different genetic backgrounds and in multiple/different environments to evaluate the efficiency of these QTLs to use in soybean breeding for selecting genotypes with a cooler canopy and drought tolerance.

## Supplementary information

Supplementary Information 1

Supplementary Information 2

Supplementary Information 3
